# Comparison of the Efficacy and Safety of Left Atrial Appendage Closure and Direct Oral Anticoagulants for Atrial Fibrillation: A Meta-Analysis of Randomized Control Trials and Observational Studies

**DOI:** 10.7759/cureus.49827

**Published:** 2023-12-02

**Authors:** Calvin R Wei, Roy Lim, Sara Khan, Syed Ali Ahsan, Mohammad Al Omari, Nima D Sherpa, Zarwa Rashid, Areeba Khan

**Affiliations:** 1 Research and Development, Shing Huei Group, Taipei, TWN; 2 Internal Medicine, Mount Sinai Hospital, Chicago, USA; 3 Medicine, California Institute of Behavioral Neurosciences & Psychology, Fairfield, USA; 4 Medicine, King Edward Medical University, Lahore, PAK; 5 Medicine, Yarmouk University, Irbid, JOR; 6 Medicine, Jahural Islam Medical College and Hospital, Bajitpur, BGD; 7 Critical Care Medicine, United Medical and Dental College, Karachi, PAK

**Keywords:** atrial fibrillation, efficacy, systematic review and meta-analysis, direct oral anticoagulant, left atrial appendage closure

## Abstract

The aim of this study was to compare the efficacy and safety of left atrial appendage closure (LAAC) and direct oral anticoagulants (DOACs) in patients with atrial fibrillation (AF). This meta-analysis was conducted as per the Preferred Reporting Items for Systematic Reviews and Meta-Analysis (PRISMA) 2020 guidelines. Two investigators performed an online database search on PubMed, Web of Science, and Scopus databases from inception to October 31, 2023, without any language or time restrictions. Outcomes assessed in this meta-analysis included all-cause mortality, cardiovascular mortality, stroke, and major bleeding events. Eight studies were included in this meta-analysis, enrolling 7,629 participants with AF (4,287 in the DOAC group and 3,342 in the LAAC group). The pooled analysis showed that the risk of all-cause mortality was significantly higher in patients in the DOAC group compared to LAAC (relative risk (RR): 1.87, 95% confidence interval (CI): 1.50 to 2.34). The risk of cardiovascular mortality was 1.60 times higher in patients receiving DOACs compared to those receiving LAAC (RR: 1.60, 95% CI: 1.12 to 2.28). The risk of stroke was not significantly different between the two groups (RR: 1.15, 95% CI: 0.95 to 1.41). In conclusion, LAAC for AF patients proves to be safe and effective for stroke prevention, exhibiting a superior profile in terms of all-cause mortality, cardiovascular events, and major bleeding compared to oral anticoagulation (OAC). These findings prompt consideration of LAAC as a preferred treatment for cardiovascular event prevention in high-bleeding-risk patients.

## Introduction and background

Atrial fibrillation (AF) represents a growing global health concern, impacting over 37 million individuals (constituting 0.51% of the world population) and exhibiting a 33% increase over the past two decades [1]. Atrial fibrillation escalates the risk of ischemic stroke by a factor of five and plays a role in approximately one-fourth of all ischemic strokes through thromboembolic mechanisms [2]. In the realm of stroke prevention for non-valvular AF, direct oral anticoagulants (DOACs) have become the favored choice due to their superior efficacy and safety compared to vitamin K antagonists [3-4]. However, it is important to note that anticoagulation remains a viable option for most patients in this population. It is noteworthy that warfarin, while generally discouraged, may be considered during the first trimester if the daily dosage is below 5mg. There exists a subgroup of patients for whom anticoagulation might not be the most suitable choice, primarily due to factors predisposing them to a significantly heightened risk of life-threatening bleeding, severe side effects, or specific conditions such as pregnancy in the first and third trimesters [3].

More recently, an alternative approach for stroke prevention in atrial fibrillation has surfaced through the use of left atrial appendage (LAA) occlusion (LAAO) or left atrial appendage closure (LAAC) [5]. Results from the National Cardiovascular Data Registry (NCDR) LAAO Registry, encompassing 38,158 procedures conducted between January 2016 and December 2018, revealed a success rate of 98.1% in achieving a leak of less than 5mm. This success was accompanied by a low incidence of major in-hospital adverse events (2.2%) [6]. As over 90% of left atrial thrombus formation occurs within the LAA [7], percutaneous LAAO has emerged as an alternative that may mitigate the bleeding risk associated with anticoagulation. Notably, for historical reasons, the two pivotal trials for LAAO utilized the vitamin K antagonist warfarin, rather than DOACs, in the comparator arm [8-9].

Nevertheless, there is a scarcity of studies directly comparing the impacts of LAAO to the novel oral anticoagulant (NOAC) therapy. As of now, the PRAGUE-17 (Left Atrial Appendage Closure vs. Novel Anticoagulation Agents in Atrial Fibrillation) trial stands as the sole prospective randomized controlled trial (RCT) that has investigated this specific comparison [10]. In light of this gap in the existing research, we are undertaking a meta-analysis to collectively analyze relevant studies and assess the effectiveness of both DOACs and LAACs in individuals with AF.

## Review

Methodology

This meta-analysis was conducted as per the Preferred Reporting Items for Systematic Reviews and Meta-Analysis (PRISMA) 2020 guidelines.

Literature Search

Two investigators performed an online database search on PubMed, Web of Science, and Scopus from inception to October 31, 2023, without any language or time restrictions. Keywords used to search for relevant articles included “left atrial appendage occlusion," “direct oral anticoagulant,” and “atrial fibrillation." The search was accompanied by synonyms, medical subject heading (MeSH) terms, and Boolean algebra operators. Additionally, a reference list of all included studies was manually screened to find additional studies relevant to the study topic.

Study Selection

Two investigators used EndNote X9 software (Clarivate, London, UK) to screen all articles retrieved through the online database search. Initially, the articles were screened using abstracts and titles, followed by full-text screening based on pre-defined inclusion and exclusion criteria. Any disagreement between the two authors in the process of study selection was resolved through a discussion. To be included in this meta-analysis, studies had to compare LAAC and DOAC in patients with AF and report at least one of the outcomes assessed in this meta-analysis. We included studies with a minimum follow-up of 12 months. We excluded reviews, editorials, and case reports.

Data Extraction and Outcomes

Two authors used a standardized data extraction form to extract relevant data from the included studies. Any disagreement between the two authors in the process of data extraction was resolved through a discussion. Using the data extraction form, the authors extracted the following data from the included studies: author name, year of publication, study design, sample size, follow-up duration, and participants’ characteristics. Outcomes assessed in this meta-analysis included all-cause mortality, cardiovascular mortality, stroke, and major bleeding events.

Data Analysis

RevMan Version 5.4.1 (The Cochrane Collaboration, London, United Kingdom) was used for statistical analysis. To compare the outcomes between LAAC and DOAC, the pooled relative risk (RR) ratio was calculated with a 95% confidence interval (CI). A cut-off of p-value was kept at 0.05. Heterogeneity among the study results was calculated as I-square (I^2^); I^2^ values of <=25% represented low heterogeneity, 25 to 75% represented moderate heterogeneity, and >75 represented high heterogeneity. A statistical test of heterogeneity was done using Cochran's Q statistics. A p-value <0.1 was considered significant for heterogeneity.

Results

**Figure 1:**
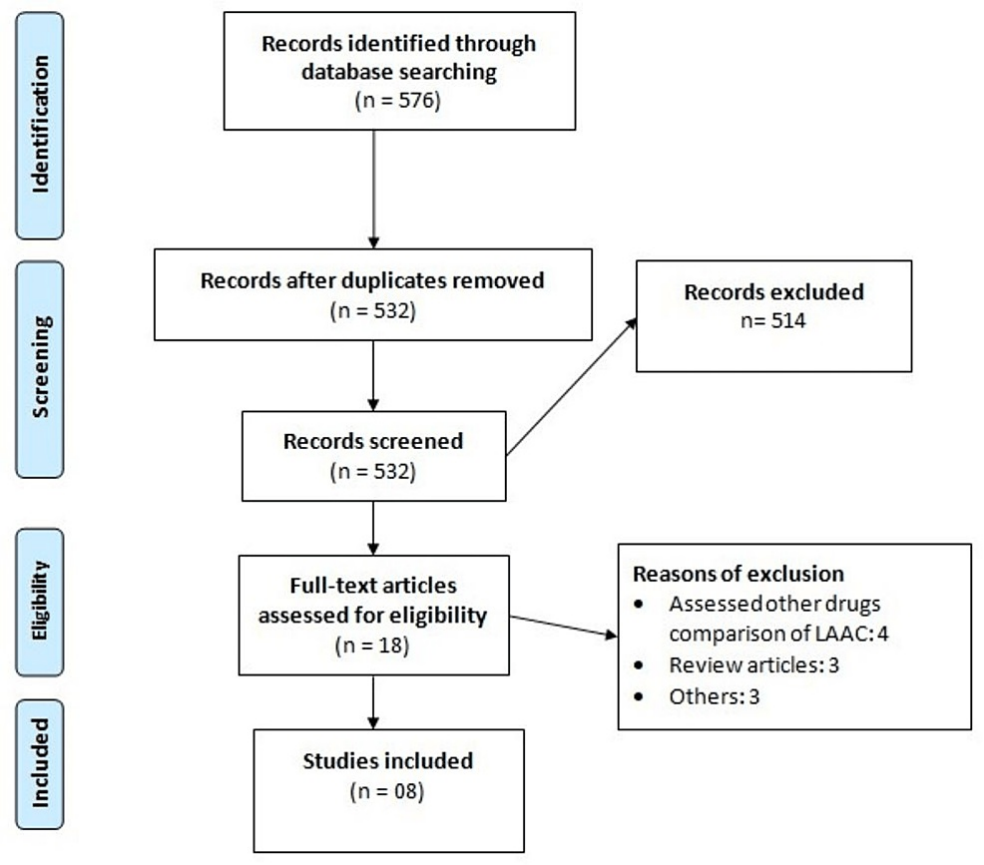
PRISMA flowchart showcasing the study selection PRISMA: Preferred Reporting Items for Systematic Reviews and Meta-Analysis; LAAC: left atrial appendage closure

The online database search yielded 576 studies. After removing duplicates, 532 studies were initially screened. The full text of 18 studies was obtained, and a detailed evaluation was done based on the aforementioned inclusion and exclusion criteria. Finally, eight studies [10-17] were included in this meta-analysis, enrolling 7,629 participants with AF (4,287 in the DOAC group and 3,342 in the LAAC group). Figure 1 shows the PRISMA flowchart, demonstrating the study selection process.

Table 1 shows the characteristics of the included studies. Among all the included studies, one was a randomized control trial (RCT) [10].

**Table 1:** Characteristics of the included studies NR: not reported; RCT: randomized control trial; DOAC: direct oral anticoagulants; LAAC: left atrial appendage closure

Author Name	Year	Study Design	Groups	Sample Size	Follow-Up	Mean Age (Years)	Males (n)	Hypertension (n)
Ding et al. [11]	2022	Observational	DOAC	661	24 months	69.2	434	455
			LAAC	661		69.9	428	462
Falasconi et al. [12]	2023	Observational	DOAC	26	40.8 months	NR	NR	NR
			LAAC	26				
Godino et al. [13]	2020	Observational	DOAC	96	24 months	NR	NR	NR
			LAAC	96				
Osmancik et al. [10]	2020	RCT	DOAC	201	19.9 months	73.2	130	186
			LAAC	201		73.4	134	186
Kei-Yan et al. [14]	2023	Observational	DOAC	1476	35.06 months	76.2	815	1004
			LAAC	874		75.5	534	586
Korsholm et al. [15]	2022	Observational	DOAC	301	24 months	76.4	202	261
			LAAC	299		76	189	232
Nielsen-Kudsk et al. [16]	2021	Observational	DOAC	1184	24 months	75.1	727	1023
			LAAC	1071		75.1	687	896
Tiosano et al. [17]	2023	Observational	DOAC	342	12 months	77.1	202	279
			LAAC	114		77.9	70	98

All-Cause Mortality and Cardiovascular Mortality

**Figure 2:**
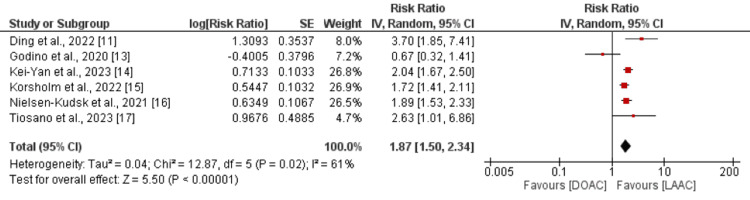
Comparison of all-cause mortality between DOAC and LAAC Sources: [11, 13-17] DOAC: direct oral anticoagulants; LAAC: left atrial appendage closure

Six studies were included in the pooled analysis comparing all-cause mortality between the LAAC and DOAC groups. As illustrated in Figure 2, the risk of all-cause mortality was significantly higher in patients in the DOAC group compared to the LAAC group (RR: 1.87, 95% CI: 1.50 to 2.34).

Significant heterogeneity was observed among the study results (I^2^: 61%, p-value: 0.02).

**Figure 3:**
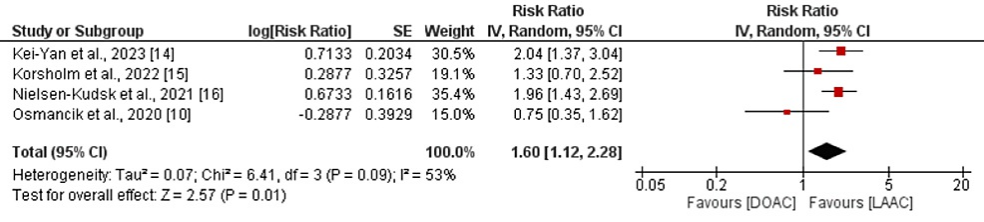
Comparison of cardiovascular mortality between DOAC and LAAC Sources: [10, 14-16] DOAC: direct oral anticoagulants; LAAC: left atrial appendage closure

Cardiovascular mortality was assessed in four studies involving 5,607 patients with AF. As depicted in Figure 3, the risk of cardiovascular mortality was 1.60 times higher in patients receiving DOACs compared to those receiving LAAC (RR: 1.60, 95% CI: 1.12 to 2.28).

Significant heterogeneity was noted among the study results (I^2^: 53%, p-value: 0.09). We conducted a subgroup analysis by excluding RCTs. The results of the pooled analysis of observational studies showed similar findings (RR: 1.89, 95% CI: 1.50 to 2.38). Heterogeneity was reduced from 25% to 0%.

Secondary Outcomes

**Figure 4:**
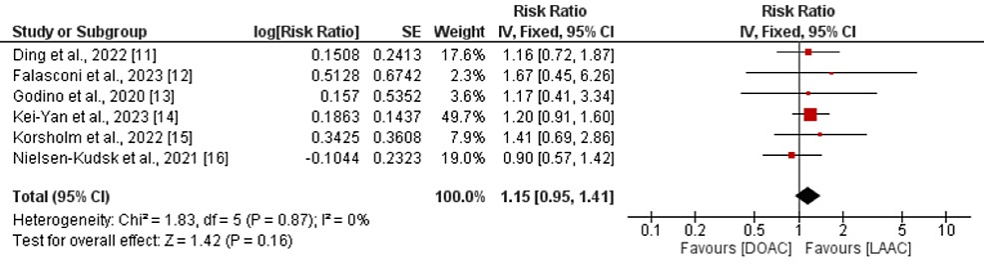
Comparison of risk of stroke between DOAC and LAAC Sources: [11-16] DOAC: direct oral anticoagulants; LAAC: left atrial appendage closure

Seven studies compared the risk of stroke between LAAC and DOAC. The risk of stroke was not significantly different between the two groups (RR: 1.15, 95% CI: 0.95 to 1.41), as shown in Figure 4.

No significant heterogeneity was reported among the study results (I^2^: 0%, p-value: 0.87).

**Figure 5:**
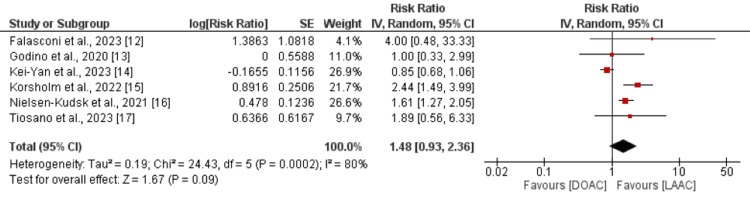
Comparison of risk of major bleeding events between DOAC and LAAC Sources: [12-17] DOAC: direct oral anticoagulants; LAAC: left atrial appendage closure

Major bleeding events were reported in six studies. As shown in Figure 5, the risk of major bleeding events was higher in the DOAC group compared to the LAAC group.

However, the difference was statistically insignificant (RR: 1.48, 95% CI: 0.93 to 2.36). Significant heterogeneity was reported among the study results (I^2^: 80%, p-value<0.001).

Discussion

Oral anticoagulation (OAC) is the recommended primary therapy for preventing ischemic stroke in AF patients [4]. Left atrial appendage closure emerged as an alternative for those intolerant to anticoagulation or desiring to avoid long-term use, demonstrating benefits over warfarin in RCTs [8, 18]. The European Society of Cardiology (ESC) guidelines suggest percutaneous LAAC only for anticoagulation contraindications [19].

In this meta-analysis of eight studies, encompassing a pooled sample size of 7,629 AF patients, stroke risk did not significantly differ between DOAC and LAAC. However, all-cause mortality and cardiovascular mortality were significantly lower in LAAC recipients compared to DOAC. Consistent with previous findings, LAAC showed similar efficacy in stroke prevention compared to OAC, even over extended follow-ups [20-21]. One RCT comparing DOAC and LAAC was included, aligning with other observational studies demonstrating reduced mortality with LAAO compared to DOAC. Of the six studies assessing this outcome, five reported a significantly higher risk of all-cause mortality in the DOAC group. The landmark randomized studies comparing LAAC with warfarin also showed long-term survival benefits and reduced nonprocedural bleeding [8].

The lower mortality associated with LAAO is likely multifactorial, with reduced bleeding from eliminating anticoagulation playing a crucial role. Major bleeding in AF patients is linked to an eight-fold increase in mortality [22], and a higher bleeding risk (HAS-BLED (Hypertension, Abnormal renal/liver function, Stroke, Bleeding history or predisposition, Labile INR, Elderly, Drugs/alcohol concomitantly) score) is associated with excess mortality [23]. Overall, bleeding reduction seems pivotal for improved clinical outcomes, including survival in AF patients. Our meta-analysis concurred with findings from randomized trials, suggesting that LAAO may enhance survival, making it an attractive alternative to DOAC, especially given the paramount importance of mortality from a patient's perspective [24]. However, more RCTs are needed to compare these therapeutic options for AF patients.

Major bleeding rates were significantly lower in the LAAO cohort compared to DOAC, implying a lower long-term bleeding risk with LAAO as a stroke prevention strategy. As patients age, comorbidities increase, raising the bleeding risk. It's speculated that longer follow-ups, beyond two years, might reveal an even more pronounced difference in bleeding rates between LAAO and DOAC [16]. However, extended follow-up data is necessary to confirm or refute this hypothesis.

While this meta-analysis provides valuable insights, it has limitations. The inclusion of only one RCT and predominantly observational studies introduces confounding factors. Non-standardized antithrombotic regimens and device-specific biases, especially considering differences between WATCHMAN™ and Amplatzer devices, contribute to potential bias. Lack of individual-level data precluded subgroup analysis. Future RCTs with longer follow-ups are essential for comparing DOAC and LAAC in AF patients.

## Conclusions

In conclusion, LAAC for AF patients proves safe and effective for stroke prevention, exhibiting a superior profile in terms of all-cause mortality, cardiovascular events, and major bleeding compared to OAC. These findings prompt consideration of LAAC as a preferred treatment for cardiovascular event prevention in high-bleeding-risk patients. Presently, LAAC is primarily proposed for those with an absolute OAC contraindication. However, whether LAAC maintains its protective role over a lifetime remains uncertain. Additionally, the absence of a standardized post-implantation antithrombotic regimen requires further investigation. Studies exploring shorter or weaker post-LAAC exposure to antithrombotic medication are warranted, potentially reducing bleeding events, especially within the first six months after implantation.
